# HIV/AIDS workplace policy addressing epidemic drivers through workplace programs

**DOI:** 10.1186/s12889-018-5072-y

**Published:** 2018-01-25

**Authors:** Bridget Chatora, Harrington Chibanda, Linda Kampata, Mutale Wilbroad

**Affiliations:** 1 Public Health Department, The University of Zambia, School of Medicine, P.O Box 5110, Lusaka, Zambia; 2 Zambia Federation of Employers, plot 6662 Mberere Road Olympia extension, PO Box 31941, Lusaka, Zambia

**Keywords:** HIV/AIDS, Workplace, Policy, Programs, Implementation

## Abstract

**Background:**

HIV workplace policies have become an important tool in addressing the HIV Pandemic in Sub-Saharan Africa. In Zambia, the National AIDS Council has been advocating for establishing of HIV/AIDS workplace policies to interested companies, however no formal evaluation has been done to assess uptake and implementation. The study aimed to establish the existence of HIV/AIDS policies and programs in the private sector and to understand implementation factors and experiences in addressing HIV epidemic drivers through these programs.

**Methods:**

A mixed method assessment of the availability of policies was conducted in 128 randomly selected member companies of Zambia Federation of Employers in Lusaka. Categorized variables were analysed on Policy and programs using Stata version 12.0 for associations: Concurrently, 28 in-depth interviews were conducted on purposively sampled implementers. Qualitative results were analysed thematically before integrating them with qualitative findings.

**Results:**

Policies were found in 47/128 (36.72%) workplaces and the private sector accounted for 34/47 (72.34%) of all workplaces with a policy. Programs were available in 56/128 (43.75%) workplaces. The availability of policy was 2.7 times more likely to occur with increased size of a workplace, *P* value = 0.0001, (*P* < 0.05). Management support was 0.253 times more likely to occur in workplaces with policy, *P* value = 0.013, (*P* < 0.05) compared to those without. Having a specific budget for programs was 0.23 times more likely to occur in workplaces with a policy (*P* < 0.05) than those without a policy. Implementation was hindered by reduced funding, lack of time, sensitisation and lack of monitoring/evaluation systems.

HIV awareness (56/56, 100%) and HIV/AIDS/Stigma (47/56, 83.93%) were the most addressed epidemic drivers through programs while Mother to Child Transmission (30/56 53.57%) and Males having sex with males were the least addressed (18/56, 32.14%).

**Conclusion:**

HIV/AIDS policies exist in the private sector at a very low proportion but policy translation was very high suggesting that workplaces with polices are likely to implement programs. The eradication of HIV/AIDS by 2030, requires addressing epidemic drivers with a focus on marginalised populations, gender integration, a wellness and rights based approach within the context of the legal framework.

## Background

The human immune deficiency virus (HIV), the virus that causes the acquired immune deficiency syndrome AIDS, continues to be one of the major causes of death globally. An estimated 36.7 million people are said to be living with HIV and AIDS globally, and 34.9 million of these are adults. Of the 1.1 million deaths attributed to HIV and AIDS in 2015, the majority, 990,000, occurred among adults [1]. Adults spend close to 60% of their time in the workplace and therefore the workplace has not been spared from the impact of HIV and AIDS.

Zambia is among the sub-Saharan African countries that have been heavily affected by the HIV and AIDS epidemic. The early years of the epidemic were marked with social and economic hardships on families and communities [2, 3] with the demise of an economically productive adult [2]. Apart from having to deal with increased deaths among employees, workplaces also faced increased production costs due to absenteeism resulting from sick leave and funeral attendance and increased medical costs [4, 5].

The National HIV and AIDS STI /TB Council (NAC) has the mandate to coordinate and monitor the national response to HIV among implementing partners in Zambia. In the last few years close to 500 organizations both in the private and public sector have been provided with technical assistance and support to develop policies and programs that address HIV and AIDS [6]. However, the extent to which these policies actually exist in the private sector is largely focused on partner reports and public sector evaluations. The prevalence of HIV in Zambia shows a steady decline from 16% to 13% [7]. This still remains high and challenges the current strategies of addressing HIV among adults towards the eradication of HIV.

The International Labour Organization’s (ILO) Code of practice on HIV and AIDS [8] and the Southern African Development Community (SADC) code of good practice on HIV and AIDS and Employment [9] are among some of the guiding principles on how workplaces should respond to HIV and AIDS [10]. While there is no legal implication for not having an HIV policy, workplaces that have them are perceived to be socially responsible and in support of HIV and AIDS programs.

The health policy initiative describes seven key dimensions [11] that interact together in a complex policy environment to either facilitate or hinder implementation, namely; 1. Policy, 2. Leadership, 3. Stakeholders, 4. Policy context, 5. Operations and Services (Programs), 6. Resources, 7. Monitoring/Evaluation processes and tools for programs. Not all workplaces with a policy will have a program as shown in a South African study that found only 41.8% (103/229) of HIV and AIDS programs [12] among workplaces with a policy. The content of HIV prevention programs can also range from simply providing condoms to a whole range of continuum including treatment to employees. An evaluation study on the mainstreaming of HIV and AIDS in line ministries in Zambia [13] found that programs ranged from 41.6 to 79.7% with a wide range of provisions and workplaces can choose to provide these as onsite, referred to government clinics [14, 15] or as an outsourced service.

An inclusiveness process of policy formulation, development, and dissemination allows for wide consultation and negotiation on policy content as well as what is feasible for the employer. Leadership for policy gives it governance, representation, responsibility, and accountability as well as advocacy for its implementation [16]. It ensures championing and planning for resources in the overall organizational plans. A survey in workplaces with a policy across Southern Africa [15] found that only 15% of union leaders had been involved in discussions during development, describing it as a ‘copy and paste’ policy, where another company’s policy was used to satisfy the requirement of having a document.

Participation in programs by both management and employees affects successful implementation. This should include people living with HIV and AIDS, as well as women. This ensures that issues affecting different groups are addressed as key stakeholders. Gender integration in HIV and AIDS programs is a key area of focus towards reducing the high female infection rates. In a study that evaluated HIV and AIDS programs in the ministry of education in Zambia it was found that low management involvement in programs [17] affected employee morale to get involved in programs. Another case study in Zambia [18] found that in spite of the widespread awareness (83.3%) of the existence of HIV programs, 63% of respondents had never even participated in the programs and mainly got involved during World AIDS day. External stakeholder involvement also necessary in ensuring linkages to key partners for services, support and resources mobilization. It also ensures that the workplace is kept abreast with new trends.

Policy decisions are usually reflected in the resources allocated for program implementation [19]. Dedicating human, time, financial, infrastructure, and material resources affect implementation based on the quantities and quality allocated. Small workplaces may have limited capacity to fund programs compared to larger workplaces [15]. A survey to assess funding sources for HIV and AIDS programs in the private sector in Zambia found that only 58% of workplaces were self-funded [20]. This limitation on resources can also extend to space, equipment, and human resources to drive the HIV/AIDS workplace policy.

An assessment of the national HIV policy implementation in Guatemala and El Salvador [11] pointed out the lack of tools for monitoring and evaluating processes for workplace programs. There was a lack of feedback which affected collaboration and the sharing of best practices, [21] making it difficult to understand what was working in policy implementation.

Emerging issues from workplaces with policies show a lack of leadership roles, a lack of involvement in policy formulation and dissemination process [16, 18], a low policy translation into programs [12] and a lack of participation in programs [18, 22] and over dependency on donor support [20, 23] for programs. Workplaces have a notable difficulty with respect to monitoring and evaluation of programs [18, 21]. There was limited current scholarly literature on private sector response to HIV in the workplace in Zambia with reports mainly focusing on public sector evaluations and partner reports.

**Fig. 1:**
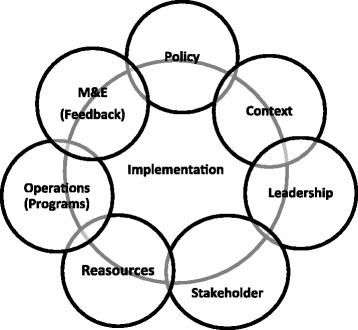
An illustration of the conceptual framework for successful implementation of HIV and AIDS workplace policy as adapted from the health policy initiative [11]. The 7 dimensions of; 1. Policy, 2. Context, 3. Leadership 4. Stakeholders, 5. Reasources, 6. Programs and 7. Monitor & Evaluations all interact in a complex policy environment to ether facilitate or hinder successful implementation of the policy

To establish the existence of HIV and AIDS workplace policies in the private sector and the extent to which programs address selected HIV epidemic drivers and to also understand the experience with implementation from the perspective of key implementers in the private sector, we asked the question, “What proportion of member companies of Zambia Federation of Employers (ZFE) in Lusaka have HIV and AIDS workplace policies and programs, and what mechanisms and resources facilitate or hinder implementation?” We used the conceptual framework in Fig. 1. This was adapted from the health policy initiative. It describes seven dimensions [11] that interact together in a complex policy environment that either facilitates or hinders successful implementation. The gaps identified in literature together with these seven dimensions for successful policy implementation formed the basis for the variables listed in Table 1.

**Table 1:** The table presents all the variables used in the study for both quantitative and qualitative data

Dependant: HIV and AIDS Policy
1. Policy: Availability: Quantitative: Yes/ No and Qualitatively: Reason for having /not having policy
A) Involvement in information for 1. Employees 2. Management; 1. Quantitatively: Yes/No 2. Extent of involvement on scale: I. Not at all, II. Limited, III. Wide, IV. Moderate, V. Do not know.
B) 1. Dissemination of Policy: Quantitative Yes/No; 2. Extent of dissemination; I. Non II. Limited with no forums, III. Wide- with forum
2. Policy content objective addressing I. Gender integration, II. Needs of people living with HIV, III. HIV and AIDS in the workplace.
Quantitative -Yes/No; Extent of addressing: I. All addressed II. Most are addressed, III. Not addressed at all, IV. No response
V. Do not know extent
Independent: Workplace Programs
3. Leadership: support for policy; Quantitative Yes/No. I. Top Management, II. Lower management, III. Union leaders.
A. Rating of effectiveness at implementation by I. Management, II.HIV and AIDS committees. Quantitative Yes /No
Qualitative explanation on experience on leadership.
4. Stake holder Involvement. Quantitative Yes/No: Qualitative: explanation on experience with stake holders.
5. HIV/AIDS workplace programs Availability: Quantitatively Yes/No
B) Employee involvement/participation in programs: Quantitatively Yes/No
I. Males, II. Females, III. Managers, IV. Union leaders, V. People living with HIV. Qualitatively explanation.
A) Elements of programs provided: Quantitative: Yes/No:
1. Awareness on HIV, 2. Voluntary Counselling service Testing, 3. Antiretroviral treatment (ART), 4. Provision of condoms Male
5. Female condoms 6. Prevention of mother to Child transmission.
B) How the workplace provides this service 1) On site, 2.Governement clinic 3. Sub contracted external provide, other.
C) Key epidemic drivers addressed by HIV Program: I) Quantitative Yes/No. II. Extent to which programs address;
I. Stigma and discrimination on HIV, II. Medical Male Circumcision and HIV, III. Condom Use and HIV IV. Sex workers and HIV
V. Alcohol abuse and HIV, VI. Gender based violence and HIV, VII. Males having sex with Males and HIV.
Qualitative explanation on extent to which selected epidemic drivers are addressed.
6. Resource: Human, Financial, Equipment, supplies and Information; Scaled sufficiency I. Completely sufficient II. Insufficient, III. Mostly sufficient, IV. Somewhat Sufficient.
7. Monitoring & Evaluation Processes: Quantitative Yes/No I. Internal, II. External monitoring 2.Qualitative explanation on Indicators and monitoring systems.

### Key terms and definitions used in the study

HIV and AIDS policy is a written document that outlines an organisation’s recognition and acknowledgment of HIV and AIDS as a workplace problem. It outlines management commitment, roles, responsibilities and position on addressing HIV and AIDS among employees. It further provides guidance on what programs the organisation is committed to undertaking to address HIV and AIDS [16]. This definition was applied for this study.

HIV and AIDS programs are action-oriented plans to prevent new HIV infections, provide care and support to all employees and manage the impact of HIV and AIDS on the workplace [13, 24]. The study used this definition.

The Workplace is a location where an employee/s provides work for an employer, usually located in a variety of settings including offices, and in any other location where work is performed [18]. We used this definition for this study.

The size of a workplace was defined by the number of employees [12]. Less than 100 employees’ defined Small workplaces, between 100 and 499 employees’ defined Medium sized workplaces. The large workplaces had between 500 and 1000 employees and the very large workplaces had more than 1000 employees.

Implementation was defined as the process of putting a decision on HIV and AIDS workplace plans into effect by systematically executing them in order to achieve desired goals and targets [25]. Therefore the presence of a program, when a workplace had a policy, was defined as implementation, irrespective of the number of programs provided.

Key implementers were defined as individuals tasked by the workplace with the responsibility of organising and coordinating the HIV and AIDS workplace programs [13].

Stakeholders are people or groups of people, affected by the outcomes, negatively or positively, or those who can affect the outcomes of a proposed intervention [26]. These include employees, employers, management, financiers implementing partners, and donors [19, 26, 27].

## Methods

### Design and sample

The study was a mixed method study design that used quantitative and qualitative methods concurrently. Lusaka was the study setting and we targeted member companies of the Zambia Federation of Employers (ZFE). Lusaka is an urban cosmopolitan town characterised by high rural /urban migrations among the working age group [6]. With a population of 1.7 million and an adult HIV prevalence rate of 20%, close to 340,000 adults are living with HIV in Lusaka alone [6]. All these factors made Lusaka an ideal setup for the study.

Study population were member companies of the Zambia Federation of Employers (ZFE), the majority of whom are private sector organisations. The study participants were men and women aged 18–59 years identified as key HIV and AIDS program implementers by their workplaces.

We used a sampling frame from the Zambia Federation of Employers (ZFE) and simple random sampling, without replacement, was used to select workplaces to be contacted. Interviews were set up by contacting the workplaces with a letter from ZFE and the National AIDS council. Appointments for interviews were set with key implementers with permission from the workplace.

To estimate the proportion of workplaces that had translated policies into programs, the sample size calculation was used: n = (z/ D) 2p (1-p) was used. At a confidence interval of 95%, CI = 0.05 and an effect size of 0.05 and a proportion for a new study set at *P* = 0.5, therefore n = (z/ D)^2^p (1-p)

$$ \mathrm{Where}:\mathrm{Z}=1.96,\mathrm{p}=0.5,\mathrm{D}=0.05 $$

$$ ={\left(1.96/0.05\right)}^{2\ \mathrm{x}}0.5\mathrm{x}\ 0.5 $$

$$ =1536.64\ \mathrm{x}\ 0.25 $$

=384.1 = 385 for a large population, since there were only 190 member companies of ZFE in Lusaka on the sampling frame, the finite population correction factor n_0_ N / n_0_ + (N- 1) was used to calculate the required sample size n.

Where sample size n = n_0_ N / n_0_ + (N- 1).Where: n_0_ is the calculated sample size without considering the finite population in the above proportion calculation.

N = the finite population = 190, which is the sampling frame from Zambia Federation of Employers. Therefore n = n_0_ N / n_0_ + (N- 1)

$$ =385\ \mathrm{x}\ 190/385+\left(190-1\right) $$

$$ =73150/574=127.4 $$

$$ =\mathrm{Initial}\  \mathrm{sample}\  \mathrm{size}=128 $$

An initial response rate of 80% among workplaces [26, 28] was anticipated from contacting 154 organisations on the sampling frame. This was adjusted to 166 workplaces at a response rate of 77.12% to obtain a sample size of 128. Purposive sampling was done for respondents that wanted to give in-depth explanation to quantitative responses already given. As a result a total of 28 in depth interviews were conducted.

### Data collection

Collection of data was done as an interviewer administered questionnaire for 45 min for both the quantitative and qualitative data. The data collection tool for both quantitative and qualitative data was designed using questions from the policy initiative implementer’s assessment tool [11] and the workplace HIV and AIDS manual [16]. This tool has been used to evaluate policy implementation in Guatemala [11] and El Salvador [21] among others. The availability of a policy document, its content, the support of management, the involvement and participation of employees and stakeholders in programs, resource sufficiency for programs as well as the processes of monitoring and evaluation for programs were all assessed quantitatively with a “Yes” or “No” response.

Likert scales were used to assess the extent to which workplace programs addressed selected key epidemic drivers of HIV and AIDS in Zambia, namely; 1) stigma and discrimination on HIV, II) alcohol abuse and HIV, III) males having sex with males and V) gender based violence (GBV) and HIV and VI) sex workers and HIV.

We also assessed the elements of HIV programs provided by workplaces, namely I) HIV and AIDS awareness, II) voluntary counselling and testing (VCT), III) male and IV) female condoms and VI) treatment for HIV and AIDS [6, 29]. We also assessed how these were provided; onsite, through government clinics or subcontracted. In-depth responses and on the implementers’ experiences were also captured using a tape recorder to provide explanations and verify transcribed data.

For validity and reliability, the questions used were adapted from the workplace HIV and AIDS policy manual [16] and from the health policy initiative implementer’s tool [11]. The National AIDS council private sector coordinator and the Zambia Federation of Employers HIV coordinator were also consulted to validate some of the questions used.

### Statistical analysis

The statistical software Stata version 12.0 was used to analyse categorized variables on leadership, resources, programs, partnerships, monitoring, and evaluation. Measures of association using Chi-Square Test - Fishers exact were employed because the contingency table outputs had cell counts less than 5 on cross tabulations as a result of the scaled breakdown responses that used in assessing the extent to which a particular variable was addressed by the programs.

Logistic regression was applied to categorical variables on policy and program. Qualitative data was put into similar topics and coded based on related topics. This was then grouped according to whether the data was coming from a workplace with a policy, a program only, both, or none. The codes allowed for a systematic identification of themes into major and sub-themes [26]. A written transcript was then generated and integrated into the quantitative data report.

Consent forms and data collection tools were kept separate and accessed only by the researcher and key stakeholders for research purposes only. Data was kept strictly confidential by the use of codes for both the company and key informants interviewed.

## Results

### Demographic characteristics

Male respondents accounted for 85/128 (66.48%) while women were 45/128 (35.15%) of the participants interviewed. The ages of respondents ranged from 25 to 58 years with an average of 42 years for both males and females. (Table 2). The majority of respondents were Human resources (67.19%) personal and the average length of stay at respondents workplace was 5 years 1 month.

**Table 2:** Demographic characteristics of the 128 key program implementers that were interviewed in 128 workplaces, distribution by gender, age and job titles

	Gender Distribution	Maximum age in years	Minimum age in years	Average Age in years	Age Range in years
Males	83 (64.84%)	56	29	42	29–56
Females	45 (35.15%)	58	25	41	26–58
Total	128			Overall average age 42 years	Overall age range 25–58 years
Distribution of Respondents by Job title in 128 workplaces surveyed.					
#	Job Title	Frequency	#	Job Title	Frequency
1	Human resources managers	86 (67.19%)	7	Deputy head teachers	2 (1.56%)
2	Human resources officer	11 (8.59%)	8	Chief executives	1 (1.56%)
3	Accountant	5 (3.91%)	9	Dean of students	1 (1.56%)
4	Assistant Accountant	3 (2.34%).	10	Receptions	1 (1.56%)
5	Manager	5 (3.91%)	11	Secretary	2 (1.56%)
6	Health and Safety officer	4 (3.13%)	12	Admin officer	2 (1.56%)
Total 128					

### Distribution of policy and programs

**Fig. 2:**
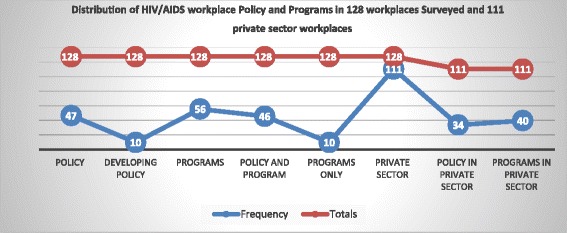
The distribution of HIV workplace policy and programs in 128 workplaces surveyed including the private sector workplaces. The distributions shows the overall proportion of workplaces with a policy, the proportion of workplaces in the process of developing their policy, the overall proportion of workplaces with programs, the number workplaces with both programs and a policy and the overall proportion of the private sector workplaces, with a policy and with programs

The proportion of private sector workplaces accounted for 111/128 (86.75%) of the total survey. The overall proportion of workplaces with a policy was 47/128 (36.71%). When it came to availability of programs, we found that 56 /128 workplaces (43.75%), had programs and that some workplaces without a formal policy also had some programs in place. A breakdown on the availability of policy and programs in the overall sample and the private sector workplaces is shown in Fig. 2.

We found that the majority of small sized organisations, 45/60 (75%) had no policies, while only 15/42 (11.72%) of the medium sized workplaces had policies. The large and very large organisations had proportionately more policies in place, 11/17 (64.71%) and 8/9 (88.89%) respectively. When size was hypothesised to influence the availability of policy, Fisher’s exact *P* = value < 0.0001 at (*P* = < 0.05) was statistically significant to suggest a strong measure of association between having a policy and the size of an organisation.

Logistic regression: on predictors of policy implementation found odds ratio at 2.7 for size of an organisation which was statically significant, *P* Value = 0.0001(*P* < 0.05). Suggesting that the odds of having a policy for workplaces in this study were increased with increased size of a workplace by 2.7 times holding all other variables constant, Table 3. Univariate analysis of top management support, funding mechanisms and having a specific budget for HIV were all statically significant when all other variables are held constant. Human resources numbers were statistically not significant with odds ratio 1.52 and *P* value = 0.24, Table 3.

**Table 3:** The determinants of implementation of HIV/AIDS workplace policy on logistic regression analysing for both univariate and Multivariate are shown

	Univariate Analysis			Multivariate Analysis		
Predictors	Odds ratio	*P* Value	95% Conf interval	Odds	*P* value	95% Confi interval
Organisation size	2.79784	0.0001*	1.740937 -4.496375	1.503014	0.430	0.5459534–4.13781
Organisation Type	0.941037	0.049*	0.8865841–0.7161456	0.8749151	0.116	0.7405965 - 1.033595
Top management	0.3003007	0.003*	.1351151 - .6674348	0.2535835	0.013*	0.0861585–0.7463523
Funding mechanism	0.15	0.014*	0.0332161–0.6773832	0.6877451	0.589	0.176609–2.678195
Specific HIV Budget	0.3223221	0.008*	0.1397287–0.7435237	0.2386781	0.027*	0.0670701–0.8493683
Human resources #	1.5258808	0.240	0.7535381–3.089546	1.719384	0.142	0.8347339–3.541585

* indicates results that show statical significance (*p*-value < 0.05) on logistic regression analysis

Multivariate analysis: Only top management support and having a specific budget for HIV and AIDS programs were statistically significant (*P* < 0.05) predictors of implementation in Multivariate analysis with odds ratio 0.25, *P*. value = 0.013, and odds ratio 0.23 *P* value = 0.027 respectively. Table 3.

Key Implementers also gave reasons for having or not having an HIV and AIDS policy: These explanations are summarised in Table 4.

**Table 4:** Qualitative summary of themes and sub themes that were generated from the interviews with key implementers in the workplace on the reasons why workplaces had an HIV and AIDS policy/program or the reasons why a workplace did not have a program or policy

Major theme	Sub-theme	Summary of findings on reasons for workplaces having policy/programs or not as reported by key implementers.
Size of workplace	Small workplace	Workplaces that were small in terms of numbers of employees, respondents reported that employees knew each other therefore they could talk freely about Health issues including HIV/AIDS there was therefore no need to have a formalised way of addressing HIV/AIDS in the workplace through programs or a policy.
		Small workplaces also reported a limitation or lack of Finances to invest in HIV/AIDS programs and policies. A few had consulted some experts, but the cost of developing an HIV/AIDS policy was not affordable for them as small workplaces
	Large workplace	HIV/AIDS was reported to be more visible among large workplace with a large number of employees. The increased burden of HIV/AIDS necessitated an organised response to HIV/AIDS in the workplace through programs and HIV policy adoption.
Type of workplace	Highly Mobility	Workplaces with highly mobile employees, the agricultural, construction and hotel industries had HIV/AIDS programs/Policy for employees.
	Core business	Workplaces such as schools, computer companies publishing and printing organisations felt that the nature of organisation did not necessitate having a policy in place.
	Religion	Religious predisposition of some organisations made it difficult in some cases to discuss HIV/AIDS as it is related to sex. This made it a taboo to talk publicly about HIV/AIDS and sex, therefore, they did not have an HIV/AIDS policy or program in place.
Health Schemes Medical insurance		Some workplaces had no HIV/AIDS workplaces policy and programs in place but had a medical scheme in place for employees which catered for general illnesses a, HIV/AIDS-related illnesses and other chronic diseases.
	Wellness approach	Some workplaces had a holistic approach to general health and safety of employees in the workplace and had therefore put in place programs focused on a wellness approach rather than programs focusing on HIV/AIDS alone.
Sensitisation	Lack of sensitization	Some workplaces did not have an HIV/AIDS policy because they did not know how to develop one and also lacked sensitisation on Multispectral response to HIV/AIDS. They admitted that having an HIV AIDS workplace policy has not been thought about.

### Policy: Content, formulation and dissemination

**Fig. 3:**
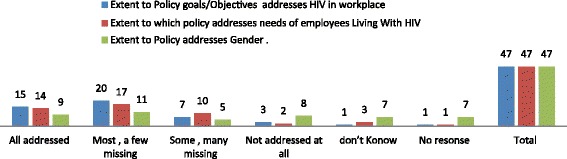
Illustrates the frequency distribution on a scaled response from key implementers on the extent to which workplace policy goals and objectives addressed 1. Gender and HIV, 2. Needs of employees living with HIV/AIDS (PLWH) and 3. HIV/AIDS in the 47 workplaces that had an HIV workplace Policies. A scale of responses from 1.all are addressed, 2. Most are addressed (a few missing), 3. Some are addressed (many missing) and 4. Not addressed at all

Content: On the extent to which policy objectives and goals addressed HIV, people living with HIV and Gender, our results showed that gender was the least addressed and that the majority of respondent’s did not knowing if their policy addressed gender at all (Fig. 3).

**Fig. 4:**
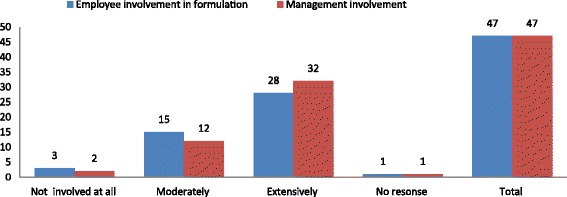
Shows the distribution of responses from key workplace implementers in 47 workplaces with a policy on the extent of participation of employees and management in the policy formulation process of their HIV and AIDS workplace Policy

Policy Formulation: The overall participation and involvement in policy formulation for management and employees was almost the same at 44/47 (93.62%) and 43/47 (91.48%) respectively. However on a scaled response we found that extensive involvement of employees was slightly lower than that of managers, while employees’ moderate involvement was higher compared to that of managers (Fig. 4).

**Fig. 5:**
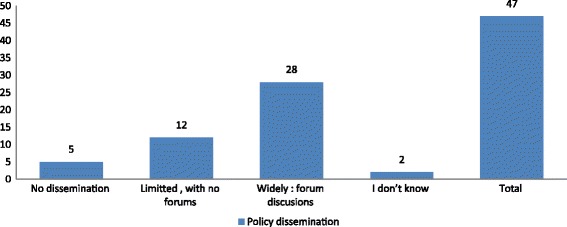
A scaled breakdown of the extent of Policy dissemination at formulation stage as reported by key Policy implementers in 47 workplaces with an HIV/AIDS workplace policy

Policy Dissemination: We found that the majority of workplaces had wide dissemination that allowed for forums of discussion, Fig. 5. Through interviews we found that workplaces used internal channels of communication such as emails, bulletins, meetings and workshops to disseminate their policy. However we also found that ongoing dissemination had reduced overtime.

### Leadership for policy and programs

Our results showed that top and lower management support for policy was almost the same, 42/57 (73.68%) and 43/57 (75.44%) respectively. However, we noted that respondents who indicated neither support nor opposition were slightly higher for lower management, 13/57 (22.81%), compared to Top management 11/57 (19.29%).Overall 36/56 (64.28%) implementers rated their management as effective at policy implementation while the rest were rated as ineffective. Qualitatively, we found that a lack of action in approving drafted policies and rebuffing negative comments from some managers on the relevance of programs was seen as a lack of support.

HIV and AIDS committees were found in 30/56 (53.57%) workplaces with programs. The majority of these, 24/30 (80%) were rated as effective at policy implementation. We found that effectiveness of committees was based on their active involvement in workplace programs.

Stake holder involvement in policy development and implementation.

External stakeholder participation in workplace programs was reported to be present in 56/56 (100%) of the workplace with programs.

We found government level stakeholders whose roles were mainly to guide and mentor workplaces on implementation, these included;

The Government through the Ministry of health, the Labour office and the District AIDS Task Force. The Zambia Institute of Human Resource Management, the International Labour Organisation, the Catholic Church and the National AIDSHIV/STI /TB council.

Another group of external stakeholders was also identified by their role in providing technical support, funding, and expertise in the management of HIV/AIDS programs, these included:

1. Afya Mzuri 2. Community HIV/AIDS Mobilization Project (CHAMP) 3. Zambia Business Coalition Association (ZBCA) 4. Zambia Health Education and communications Trust (ZHECT,) and 5. Zambia AIDSlaw Research and Advocacy Network (ZARAN) [20] to name a few.

We found that implementing partners were well coordination and transparent in implementation under Zambia workplace partnership. Mutual respect among the partners was reported to exist because these organizations are not in competition but work together to address the HIV and AIDS epidemic in Zambia.

### Addressing elements of HIV and AIDS through programs

We found that HIV and AIDS awareness was being addressed by all workplaces with programs 56/56 (100%) and that the majority of workplaces, 53/56 (94.0%) provided voluntary counselling and testing services. However, female condoms were noticeably absent in twice as many workplaces compared to male condoms (Table 5). Some workplaces provided free HIV treatment to employees through their own clinic, a government clinics, an external provider or through a medical insurance schemes (Table 5).

**Table 5:** The table shows how workplace programs address and provide elements of HIV awareness, voluntary HIV counselling & testing, provision of male and female condoms and the provision of antiretroviral treatment to employees in 56 workplaces with programs. As reported by key workplace program implementers in 56 workplaces with HIV programs. Fisher’s exact test of measure of association between having a policy and proving the program elements is also shown

Element of Program	Onsite	Gov clinic	Medical Insurance	Out Sourced	Not provided	Fishers exact
Awareness	42 (75%)	3 (5.36%)	8 (14.29%)	3 (5.36%)	0 .00	0.807
VCT	20 (35%)	12(21.42%)	12(21.42%)	9 (16.07%)	3 (5.37%)	0.614
Female Condoms	26 (46.42%)	1 (1.78%)	0.0	5 (8.92%)	24 (42.85%)	0.197
Male Condoms	38 (67.85%)	12(21.4%)	0.0	5(8.92%)	12 (21.42%)	1.000
ARVS	4(7.14%)	14 (25.0%)	24 (42.85%)	9 (16.07%)	5 (8.92%)	0.271

For all selected elements of HIV and AIDS programs provided in the workplaces, Fisher’s Exact test was not statistically significant (*P* > 0.50) to suggest a relationship between having a policy and provision of selected program elements.

### Addressing selected key HIV and AIDS epidemic drivers

**Fig. 6:**
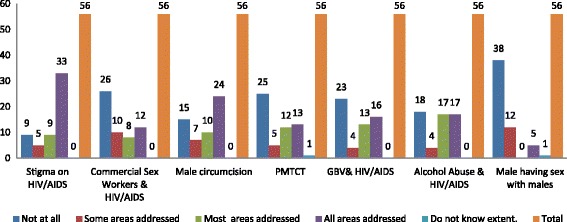
Shows the extent to which some key selected HIV/AIDS epidemic drivers are addressed through workplace programs as reported by key policy/program implementers in 56 workplaces with programs and policy

The extent to which selected epidemic drivers were addressed is illustrated in Fig. 6. Sex workers and HIV as an epidemic driver were a concern in the agricultural sector and in workplaces that reported frequent traveling of their employees. Most respondents reported that they had difficulty in addressing this because it is a personal choice, as explained by one implementer,

*“Especially that our employees travel a lot away from home some people may be used to having sexual relations regularly with their partners, therefore when they travel they have it with Sex workers so we do try to talk about it during sensitisation talks and during condom demonstration”* ^***KIHp4***^ ***.***

The prevention of mother to child transmission (PMTCT) and HIV as an epidemic driver was addressed in 30/56 (53.57%) workplaces with programs. We found that this was integrated in talks on HIV and AIDS awareness and prevention. However, workplaces providing medical health insurance for their employees felt that PMTCT was adequately covered in the health insurance scheme and there was no need to address it as a workplace.

Gender Based Violence (GBV) was not addressed at all in 25/56 (44.64%) workplaces. It was integrated in sexual harassment policies and some activities that correspond with national and international statutes on GBV. Some workplaces were also partnering with the victim support unit of the police and with gender experts in on GBV.

Alcohol abuse and HIV**,** was a concern among 33/56 (58.92%) workplaces. Many workplaces addressed it alongside GBV and condom utilisation as a cause of condom failure. We found that faith-based organisations, the drug enforcement unit of the police were among the partnerships that workplaces used to educate employees on alcohol and drug abuse. Through interviews we found that alcohol abuse affected productivity and safety in a number of workplaces, as explained by one implementer,

*“We discuss it* (Alcohol Abuse and HIV) *a lot because of the industry we are in, we have had quite a few issues with alcohol abuse among some of our employee’s*. E*specially during those days when tujilijili* ( local name for packaged alcoholic spirits )*were common…. and we were in trouble with alcohol abuse and employees being drunk on duty*” ^*KIHp*^*.*

Voluntary Medical Male Circumcision (VMMC) and HIV**:** was addressed by the majority of workplaces 41/56 (73.21%) and the actual service was refereed to expert providers and government clinics. We also found that the use of champions on Male Circumcision was a key strategy in promoting voluntary male medical circumcision.

Males having Sex with Males and HIV: was not addressed at all in 38/56 (67.86%) workplaces. Key implementers explained that this is because it is illegal in Zambia and not openly practiced. Some key implementers said it was also an uncomfortable subject because culturally it is perceived as a taboo and immoral, one implementer said,

*“We have never talked about this* (Males having sex with males and HIV)*. We know that it may be happening but due to cultural norms it’s not only a taboo but illegal it’s a topic we hardly talk about. Such a person here would suffer because 90% of our employees are males and heterosexual and are married. There are no known males having sex with males here”* ^***Kip****3*^*.*

### Participation in HIV AND AIDS workplace programs

**Fig. 7:**
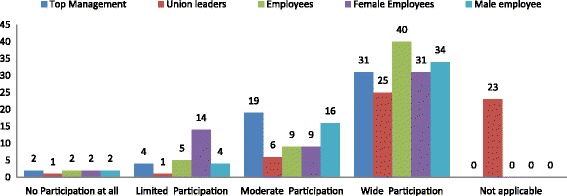
Extent of participation in programs for I. Top management, II. Union Leaders, III. Employees IV. Female employees and V. Male employees in 56 workplaces with HIV/AIDS workplace programs as reported by key implementers of HIV/AIDS workplace programs

The extent of participation in programs by managers, union leaders, and employees is illustrated in Fig. 7. Employees had wider participation compared to Top management. However we found that there were fewer females with moderate and wide participation compared to males. We also noted that moderate participation of males was more compared to that of females. A test of independence using Fishers exact to compare male and female participation found *P* value = 0.0001. Suggeting that workplaces with a policy were more likely to have more female participation in workplaces programs. This was statistically significant at (*P* < 0.05).Through interviews, we found that low participation of female employees in workplaces programs was attributed to cultural factors as female employees were generally reported to be shy when it came to talking openly about issues relating to sex and HIV and AIDS.

Resource for Policy /Program Implementation. (Financial, Human, Infrastructure and equipment, Information).

**Fig. 8:**
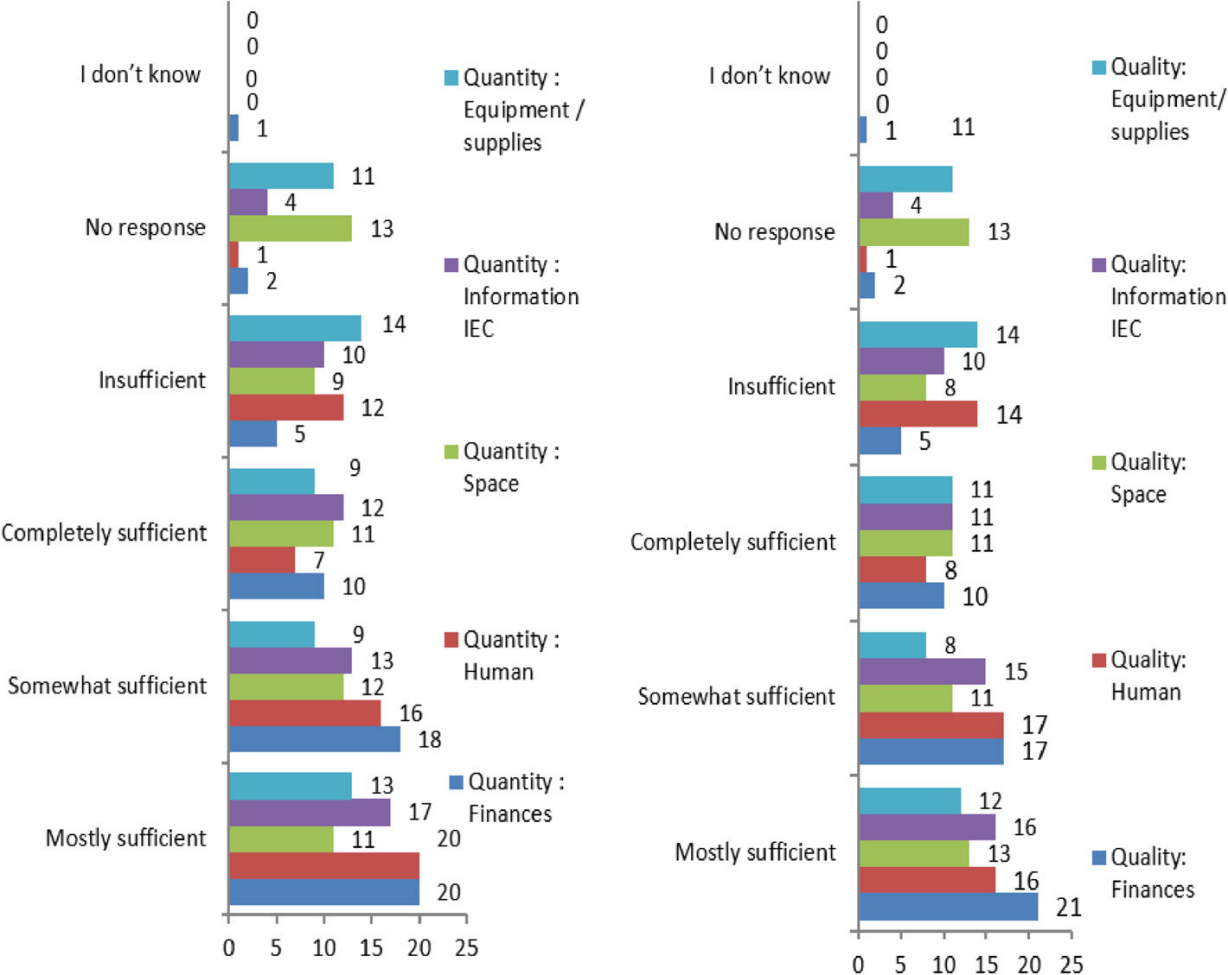
The extent of sufficiency of resources (both Quantity and Quality) allocated for HIV programs as reported by key implementers in 56 workplaces with programs: 1. Finances 2. Human Resources 3.Infrastructure Space 4. Equipment and supplies and 5.Information

The extent of sufficiency of resources (finances, equipment/ supplies, information, and human resources) for HIV programs, is illustrated in Fig. 8.

Financial resources: We found 28/56 (50%) workplaces with a specific budgets for HIV programs. Fishers exact test at *P* value = 0.012 showed that workplaces with a policy were more likely to have a budget specific for HIV and AIDS programs and statistically significant (*p* < 0.05) to suggest that policy influenced having a specific budget for HIV programs.

We found that some barriers to accessing funding for HIV and AIDS programs among most workplaces included; 1. donor funding being tied to specific activities; 2. Some donors were winding up their own activities and as well as their support for HIV programs; 3. Some workplaces also had competing operational needs that took priority for finding even when there was an allocated budget for HIV programs; 4. Some workplaces also experience delayed and reduced allocation of funding from their donors. As a result of these challenges most of the workplaces reported having to reduce the number of planned activities for their HIV programs.

Human Resources: This was reported as sufficient in 42/56 (75.0%) workplaces and insufficient in 14/56 (25%). Fisher’s exact test for association between human resources numbers (quantity) and availability of policy in workplaces with programs was significant at *P*-value 0.003, (*P* < 0.05) to suggest that availability of Policy influenced Human resources for programs and therefore the implementation of policy.

Resources Infrastructure / Space for programs: This was found to be sufficient in 34/56 (60.71%) and the quality of space (privacy) was sufficient in 35/56 (62.50%). In-depth interviews showed that majority of workplaces with programs had adequate space and infrastructure to carry out program activities, where this was limited implementers were able to find some space within the workplace.

Equipment Supplies: We found insufficiencies in the provision of female condoms and that female condoms were unavailable in most workplaces. The in-depth interviews revealed that some equipment insufficiencies were due to tear and wear on vehicles and utilisation of out-dated equipment such as videocassette recorders. We also learnt that most training materials were not current with the changing needs of people living with HIV and AIDS.

Information Resources: Through interviews we found that information materials were obtained through partnerships and that some reported insufficiencies were as a result of partners ending their support. Some implementers also reported burnout with HIV and AIDS-related topics and wanted of information on health in general and other illnesses, which was limited.

### Monitoring and evaluation for policy/programs

The monitoring of HIV and AIDS programs was done in 44/56 (78.57%) workplaces and only 19/56 (33.93%) of them had an external monitoring organisation and 2/56 (3.57%) did not know if they had one. In contrast, internal monitoring was present in 42/56 (75.00%) workplaces.

Through explanations from key implementers we found that overall indicators were ill-defined and inconsistent which made it difficult for reporting and monitoring program outcomes.

## Discussion

The study established that the existence of HIV and AIDS policies in the private sector was low. The availability of policy was influenced by the size of organisation and sensitisation on response to HIV and AIDS. Whereas the public sector is mandated to respond to HIV and AIDS [30, 31] this is not the case with the private sector. Which may explain larger proportions of policy found in other studies for example, 81.81% (18/22) in line ministries in Zambia. Prevalence across sub-Saharan Africa workplaces [13, 24] found between 41 and 89% with more policies in larger workplaces compared to small. While we did not take into account sampling by size and type of workplace, qualitative interviews agreed with the conclusion that the size of a workplace influenced policy availability. When implementation was based on the total sample size, our findings are consistent with the public sector findings in Zambia where programs ranged from 41.45%–68% [18, 28].

The low Management participation in programs compared to that of employees was confirmed through qualitative interviews. This is similar to findings in the Ministry of Education [18] with results as low as 25–30% (*n* = 64). Utilisation of scales strengthened the findings that employees had wider participated in programs compared to top management. This is contrary to findings in line ministry [13] where there were no significant differences in participation between management and general workers. The lack of policy support by keeping policies in a draft [18, 20, 28] restricts access to resources however expert consultants can be used [20] in some cases.

Given the higher prevalence of HIV among women [32] compared to men, coupled with the evidenced paucity in female-driven HIV prevention tools and the cultural limitations of female participation in programs, the limited gender integration [16] continues to undermines progress on eradicating HIV and AIDS and needs to be strengthened.

The public sector has a 10% budgetary allocation [13, 30, 31] towards HIV programs, which may not be feasible in the private sector, evidenced by only half of the workplaces with a specific budget for HIV programs. This is consistent with an earlier study where only 58% of workplaces had self-funded programs [33]. Strategic ways of financing programs to reduce donor dependencies [20] such as using medical insurances schemes [6] and less costly health promotion and prevention strategies should be explored.

Past studies have shown provision of VCT in workplaces between 48.30% [18] and 75% [24], with the majority being onsite this has increased uptake compared to offsite alternatives [34, 35] because of the convenience of access [29] frequent awareness and group nature of campaigns [36]. As a useful way of managing costs and also avoiding Stigma [37, 38] and lack of confidentiality in small workplaces, medical insurance schemes and referral to government clinics were used to provide VCT services [24, 39]. Provision of ART services was high, contrary to past surveys where only 50% and less [24] of employers were providing ART, bearing in mind a time difference in the limitation of access to ART. However a more recent study found access to ARVS through workplaces at 40.09% (123/301) with a strong association to HIV programs [18].

### Selected drivers of the epidemic

Homosexuality and its risk factors to HIV were ill addressed in workplaces programs. To avoid missing hot spots towards eradicating HIV and AIDS and because of the higer risk of HIV transmsion involved, there is need to address homosexuality [40] within the context of the legal framework [1]. This, together with Gender based violence, PMTCT, and alcohol abuse, were all poorly addressed. Violence against women has been closely linked to HIV transmission [41, 42] as it limits negotiation of safe sex [43, 44]. In Zambia women (88.8%), [32] are more aware than men (82.1%) [32], about HIV transmission through breastfeeding [32] and more aware (82.0%), that this risk can be reduced during pregnancy [45], compared to men (65.8%) [32]. The workplace provides an environment to educate both men and women and increase partner involvement and also provide linkages for support on GBV. Similarly, risky sexual behaviours such as unprotected sex, sex with a stranger [17] and paying for sex [27, 46], which are all associated with alcohol abuse and HIV and AIDS [36], therefore need to be incorporated in HIV workplace programs.

The National Aids reporting format for monitoring and evaluation (M/E) has standardised indicators and a reporting format for HIV and AIDS programs [47], yet there was a lack of well-defined M/E systems. Similar findings emerged from other studies [13, 15, 18] were the lack of tools was found to have reduced coordination [5, 21] and limited local level partnerships. Lack of access to tools makes it difficult to collect data, in turn compromising the quality of data and decision making and planning of evaluations on program impact [19].

## Limitations of the study and methodological considerations

There was a limitation on academic literature on HIV and AIDS activities in the private sector in Zambia which relied largely on partner reports and research from the public sector. Sampling by size and type of workplaces was not employed, therefore caution should be applied to associations and comparisons of findings. However, the qualitative findings supported most of the associations made. A sampling frame of ZFE members companies in Lusaka was used to obtain a proportionately large number of private sector workplaces, therefore limiting the generalisation of results. There may have been some over reporting on workplace provisions for policies form key implementers, however detailed questioning on sufficiency’s and extent of coverage as well as utilisation of scales showed the adequacy and challenges of implementation.

There was a large number of variables in the study, while this may compromise the strength of measures applied, the utilisation of a mixed method and scaled responses provided some cross checking. A methodological weakness to consider was the utilisation of any form of prevention elements to indicate implementation. However using scales to examine the extent to which programs addressed key drivers, provided additional strength in assessing implementation. The tool also provided for response options when the respondent could not provide a response.

A broader perspective from managers, policy makers and other stakeholder’s was not included.

The utilisation of health insurance services in managing HIV and AIDS and other chronic diseases through workplace programs was not explored further. This is a focus for further research towards a sustained financing of health-related workplace programs.

## Conclusions

The majority of workplaces surveyed were from the private sector, this means that the private sector workplaces had a low response to HIV and AIDS through Workplace policy adoption but there was a very high proportion of implementation of HIV and AIDS programs wherever a policy was available.

The study concluded that Workplace programs addressed some epidemic drivers poorly. There was a need to strengthen gender integration with a targeted approach on GBV, PMTCT, males having sex with males and a wellness approach to HIV programs within the context of the legal framework.

The study results contribute towards informing policy makers on the extent of private sector response to HIV and AIDS and the challenges experienced in implementation of these programs. The results are useful for planning of resources for programs and approaches aimed at addressing key epidemic drivers of HIV and AIDS.

## Abbreviations

AIDS: Acquired immunodeficiency syndrome
GBV: Gender based violence
HIV: Human immunodeficiency virus
ILO: International Labour Organisation
M/E: Monitoring and evaluation
NAC: National HIV and AIDS /STI/ TB Council
PLWH: People living with HIV
SADC: Southern African Development Community
STI: Sexually transmitted diseases
W.H.O.: World Health Organisation
ZFE: Zambia Federation of Employers
